# Association between Workplace Violence and Depressive Symptoms among Primary Healthcare Professionals in Shandong, China: Meaning in Life as a Moderator

**DOI:** 10.3390/ijerph192215184

**Published:** 2022-11-17

**Authors:** Meiqi Wang, Haipeng Wang, Zhen Wei, Yifan Wang, Long Sun

**Affiliations:** 1 Centre for Health Management and Policy Research, School of Public Health, Cheeloo College of Medicine, Shandong University, Jinan 250012, China; 2 NHC Key Laboratory of Health Economics and Policy Research, Shandong University, Jinan 250012, China

**Keywords:** workplace violence (WPV), meaning in life, depressive symptoms, primary healthcare professionals, Shandong, China

## Abstract

Background: Workplace violence (WPV) is common in healthcare settings. However, the association between WPV and depressive symptoms has not been explored among primary healthcare professionals, especially in China. The assumption of the moderating effort of meaning in life on the association has also not been tested. The purposes of the study are to investigate the relationship between WPV and depressive symptoms and identify the moderating role of meaning in life among primary healthcare professionals in China. Methods: In this study, we collected 2530 valid questionnaires. The participants were composed of primary healthcare professionals in Shandong province, China. WPV, meaning in life (including presence of life and search for life), depressive symptoms, and some social-demographic variables were evaluated. The SPSS macro was used to build the moderating relationship. Results: WPV was positively correlated with depressive symptoms (β = 9.09, *p* < 0.001), and meaning in life was negatively associated with WPV and depressive symptoms (β = −0.13, *p* < 0.05) among primary healthcare professionals in China. For primary healthcare professionals with low presence of life, presence of life aggravated the relationship. Conclusions: The current study has verified the association between WPV and meaning in life, and the relationship between WPV and depressive symptoms has been supported among primary healthcare professionals in China. Meaning in life and presence of life as moderators play crucial roles in the relationship between WPV and depressive symptoms.

## 1. Introduction

Workplace violence (WPV) is defined as “incidents where staff is abused, threatened, or assaulted in circumstances related to their work” [1]; it has two main types, one is physical violence, another is psychological violence, including verbal abuse [2]. WPV is common in healthcare settings and, in China, a quarter of WPV relates to healthcare professionals [3]. In other countries, WPV in healthcare systems is widely prevalent [4,5]. Primary healthcare professionals have large workloads that include prevention, medical health, rehabilitation, health education, and promotion and family planning services. It means that the contact frequencies between primary healthcare professionals and patients are more frequent than other healthcare professionals, which also influences the odds of WPV. There are insufficient resources and lack of personnel in the basic medical institutions, leading to arouse dissatisfaction in patients, which in turn induces incidences of WPV [6]. A cross-sectional study found that 51.64% of community health professionals experienced WPV in Guangzhou and Shenzhen [7]. A study showed that 82% of primary health workers were exposed to verbal abuse, and 74% of primary healthcare professionals were exposed to physical violence [8]. One study estimated the prevalence of psychological violence at Chinese township hospitals and they found that 38.69% of healthcare professionals experienced verbal abuse in their careers, and 48.72% of the perpetrators were the relatives of patients [9].

Previous studies also investigated the persistently negative effects of WPV on healthcare professionals, such as physical, psychological, emotional, work functioning, and care quality aftermaths [10]. A big data study based on the media reports from 2000 to 2015 in China revealed 290 cases in terms of injury or the death of healthcare workers caused by WPV [11]. Abundant studies have supported the association between WPV and negative mental health among healthcare professionals [12,13]. Some scholars identified that healthcare professionals who experienced WPV were more likely to develop depression [10,14]. Experiencing WPV eliminated job satisfaction and meaning in life, decreased healthcare work efficiency, and even elevated the levels of depression, job stress, job burnout, and turnover intentions [2,15], which had an impact on patient health. A few foreign studies proved that primary healthcare workers who suffered violence presented with depression [16]. Nevertheless, domestic studies have not investigated the conclusions among primary healthcare professionals. Because of the large population, primary healthcare professionals in China and abroad have various work contents, workloads, job satisfaction, and social status, and the former confound more job stress and violence during the work. In China, there are various job contents and workloads between primary healthcare professionals in primary healthcare settings and healthcare professionals in public hospitals. Therefore, the association between WPV and depressive symptoms among primary healthcare professionals in China needs to be verified.

Meaning in life is absolutely necessary for a person’s life. Meaning in life is the “sense made of, and significance felt regarding, the nature of one’s being and existence [17]”. Primary healthcare professionals who have experienced WPV may think that they are meaningless to others, they have a meaningless job, and even a meaningless life. It is worth mentioning that meaning in life comprises two dimensions: presence of life and search for life; many studies have reflected that the former is negatively associated with the latter [18,19]. Presence of life represents the extent to which life is considered meaningful, while search for life represents the impetus to search and discover meaning in life [20]. It means that people who have high meaning in life do not search for meaning in life; only those who have low meaning in life desire to search for meaning in life. However, a few studies have had contrary conclusions that presence of life and search for life have a positive correlation [21]. Some people who have substantial meaning in life may consequently search for meaning [22]. The previous studies have explored the positive relationships between meaning in life and health-protective outcomes [22,23]. Studies have indicated that presence of life and search for life have significant protective impacts on mental health, such as depression [24,25]. Likewise, the negative associations between meaning in life and all kinds of physical and psychological problems have been demonstrated [26,27]. Even a low loss of meaning in life is associated with physical and mental health [28].

Victims who have experienced traumatic events may perceive themselves, others, or the world as unpredictable and unsafe, increasing their negative beliefs [26,29]. When primary healthcare professionals are faced with WPV, those who embrace higher meaning in life may be less influenced and more likely to recover from depressive symptoms than those who embrace lower meaning in life. Few studies have explored the association between WPV and meaning in life. However, the relationship between WPV and depressive symptoms has been verified and meaning in life is a component of depressive symptoms. Then, we have reasons to investigate the association between WPV and meaning in life. So far, the moderating mechanism in the relationship between WPV and depressive symptoms is still unclear. Meaning in life may be a vital, underlying mechanism in the relationship.

The findings of previous studies have shown the associations between depressive symptoms and either WPV or meaning in life [24,30]. However, few studies have investigated the correlation between WPV and depressive symptoms among primary healthcare professionals in China and the moderating role of meaning in life. There are fewer primary healthcare professionals per capita in China than that in other countries. Additionally, they have huger workloads, serve more residents, and utilize older equipment than other healthcare professionals in China. They are prone to experience WPV and have more depressive symptoms. It is necessary to investigate the relationship between WPV and depressive symptoms among primary healthcare professionals in China.

Therefore, the purposes of the current research are three-fold: (1) to ensure the associations between WPV and meaning in life (including presence of life and search for life); (2) investigate the relationship between WPV and depressive symptoms among primary healthcare professionals in China; (3) identify the moderating roles of meaning in life (including presence of life and search for life) between WPV and depressive symptoms among primary healthcare professionals in China. The findings, to some extent, will be significantly helpful for the government and administrators to develop valid intervention projects, lessening the prevalence of WPV and depressive symptoms caused by WPV among primary healthcare professionals in basic or township medical institutions.

## 2. Materials and Methods

### 2.1. Participants and Interview Procedure

In this study, we collected 2530 valid questionnaires. The participants were composed of primary healthcare professionals in Shandong province, China. We utilized the multiple stratified random cluster sampling method. Firstly, we randomly chose five municipalities (Zaozhuang, Liaocheng, Zibo, Weifang, and Qingdao) from 16 cities according to the level of GDP in Shandong, China [31]. Next, we randomly selected one county from each municipality (Taierzhuang, Gaotang, Yiyuan, Qingzhou, and Huangdao), and all the primary medical institutions were selected from the five counties. All of the primary healthcare professionals working on the survey date were required to participate in the study.

Before the data collection, the survey procedures were approved by the Institutional Review Board of Shandong University School of Public Health. Data in this cross-sectional study were collected through self-reported questionnaires from October 2020 to December 2020. The interviewers were trained postgraduate students and understood the research and questionnaires prior to the beginning of the study. To this end, at least two trained students examined the contents of the questionnaires, and the questionnaires with missing or unclear data should be revisited and refilled. All participants were volunteers and completed written informed consent forms.

### 2.2. Materials

The demographic information of participants was collected, including age, gender, religious belief, working years, working days/week, education, major, budget post, nature of job, job types, and income level. Age was calculated using participant date of birth. Gender was defined by male (1) and female (2). Religious belief was assessed by Yes (1) and No (2). Working years and working days/week were filled out by the participants. Education was assessed as High school and above (1), Technical school (2), and Technical secondary school and below (3). Major was evaluated by Clinical medicine (1) and others (2). Budgeted post was assessed by Yes (1), No (2) and Unknown (3). Nature of job was assessed by Township hospitals (1) and Village clinics (2). Job types were assessed by Medicine (1), Nursing (2), Public health (3), and Others (4). Income level was assessed by Higher (1), Average (2), and Lower (3).

The data regarding WV were obtained using the question, “Have your patients or their relatives ever done the following to you?” and the participants could choose options of “verbal abuse, physical assault, both, none”. According to the answers, we combined four answers to two answers, Yes (1) and No (2). “Yes” expressed that primary healthcare professionals had experienced WPV, and “No” represented that primary healthcare professionals had not experienced WPV perpetrated by patients or their relatives.

Meaning in life was assessed by using the Meaning in Life Questionnaire (MLQ; [17]). The MLQ was a 10-item self-administrated measure rated on a 7-point Likert ranging from Strongly disagree = 1 to Strongly agree = 7. The total score was the sum of 10 items (one item was inversely scored), leading to a score range of 10 to 70; the higher score represented the higher meaning in life among the primary healthcare professionals. There were two subscales, the presence of life subscale (e.g., “I understand my life’s meaning.”) and the search for life subscale (e.g., “I am looking for something that makes my life feel meaningful.”). The internal consistency coefficient alphas of the total scale, presence subscale, and search subscale are, respectively, 0.861, 0.738, and 0.896 in this study.

Depressive symptoms were measured by utilizing the Center for Epidemiologic Studies Depression Scale (CES-D Scale; [32]). The CES-D scale is a brief 20-item self-report measure, and each item is rated on a four-point Likert from 0 = Within 1 day to 3 = Five to Seven days. The final score was the sum of all items (four items were reversely scored), ranging from 0 to 60. The sample item was as follows “I was bothered by things that usually do not bother me”. The higher score reflected more frequencies of depressive symptoms during the past week. In the current study, the internal consistency coefficient alpha is 0.89 within these data.

### 2.3. Data Analysis

All statistical analyses were performed using SPSS, version 23.0. We used descriptive statistics to analyze the means and standard deviations for continuous varieties, numbers, and percentages for categorical variables. A one-way ANOVA or Chi-square test was conducted to assess the differences for the continuous and categorical variables across one group that experienced WPV and another group which experienced no WPV. Linear regression was used to study the variables related to depressive symptoms controlling the covariates and the interaction between WPV and depression. The SPSS macros (PROCESS v3.5 by Andrew F. Hayes) were used to build the moderating relationships between WPV, meaning in life (presence of life and search for life), and the depressive symptoms controlled for demographic variables and meaning in life (presence of life and search for life), as moderators with Model 1. The categorical variables were translated into dummy variables. WPV and depressive symptoms were, respectively, added to the Y variable and X variable, and the control variables were added to the Covariates. All significance tests were two-tailed, and a threshold of *p* ≤ 0.05 indicated statistical significance.

## 3. Results

### 3.1. Descriptive Statistics

The descriptive statistics, including social-demographic variables, independent variables, moderating variables, and dependent variables, were shown in Table 1. The study was conducted using 2530 participants ranging in age from 19 to 104 years, 1089 (43.0%) males and 1441 (57.0%) females (Mean = 41.55, SD = 0.80). One-third of the primary healthcare professionals graduated from technical schools. In the study, 57.4% of the primary healthcare professionals worked at township hospitals, and 42.6% of the primary healthcare professionals worked at village clinics. The findings showed that 14.9% of the primary healthcare professionals experienced verbal abuse, 3.1% of the primary healthcare professionals experienced physical assault, and 2.3% of the primary healthcare professionals experienced both of the above types perpetrated by patients or their relatives. In total, 15.7% of primary healthcare professionals experienced WPV caused by patients or their relatives. More detailed information is provided in Table 1.

The results indicated that WPV was associated with age (F = 29.372, *p* < 0.001), working years (*χ*^2^ = 42.252, *p* < 0.001), working days/week (F = 38.037, *p* < 0.001), education (*χ*^2^ = 63.424, *p* < 0.001), major (*χ*^2^ = 8.522, *p* < 0.01), budget post (χ^2^ = 10.462, *p* < 0.01), the nature of the job (*χ*^2^ = 9.273, *p* < 0.01), income level (*χ*^2^ = 7.513, *p* < 0.05), depressive symptoms (F = 55.765, *p* < 0.001), meaning in life (F = 8.292, *p* < 0.05), and presence of life (F = 12.533, *p* < 0.001). Primary healthcare professionals with lower age, fewer working years, and fewer working days every week, higher education, non-clinical major, budget post, working at village clinics, and lower income level than their colleagues were more likely to experience WPV due to patients and their relatives. WPV was not correlated with gender (*χ*^2^ = 0.393, *p* > 0.05), religious belief (*χ*^2^ = 0.792, *p* > 0.05), job types (*χ*^2^ = 3.543, *p* > 0.05), or search for life (F = 2.658, *p* > 0.05). Primary healthcare professionals who encountered WPV were more depressive and had less meaning in life than those who did not encounter WPV due to patients and their relatives.

### 3.2. Linear Regression Analysis for Depressive Symptoms among Primary Healthcare Professionals

Model 1 in Table 2 represented the variables associated with depressive symptoms, with Linear regression controlling the covariates (R = 0.378, R^2^ = 0.143, F = 23.185, *p* < 0.001). Depressive symptoms were associated with WPV (β = 3.11, *p* < 0.001, 95%CI = 2.12, 4.11) and meaning in life (β = −0.27, *p* < 0.001, 95%CI = −0.30, −0.23). Females were further associated with depressive symptoms compared to males (β = −2.00, *p* < 0.01, 95%CI = −3.12, −0.86), and low-income level was also associated with depressive symptoms compared to an average level of income (β = 2.73, *p* < 0.001, 95%CI = 1.91, 3.55).

After controlling the interaction and covariates in Model 2, depressive symptoms were associated with WPV (β = 9.09, *p* < 0.001, 95%CI = 4.26, 13.91), and meaning in life (β = −0.13, *p* < 0.05, 95%CI = −0.24, −0.02). The interaction between WPV and meaning in life had a significant effect on depressive symptoms (β = −0.12, *p* < 0.05, 95%CI = −0.21, −0.02). Females were further associated with depressive symptoms compared to males (β = −1.95, p < 0.001, 95%CI = −3.08, −0.83), and low-income level was also associated with depressive symptoms compared to average level of income (β = 2.71, *p* < 0.001, 95%CI = 1.89, 3.53). The correlations between depressive symptoms and other variables were nonsignificant. As expected, the results corroborated meaning in life and moderated the relationship between WPV and depressive symptoms. The interactive relationship explained 14.5% of the variance in depressive symptoms.

Table 3 represented the associations between presence of life, search for life, and depressive symptoms. Depressive symptoms were associated with presence of life (β = −0.72, *p* < 0.001, 95%CI = −0.79, −0.67) in Model a, and search for life (β = −0.18, *p* < 0.001, 95%CI = −0.23, −0.12) in Model c, controlling the covariates. After controlling the interactions, presence of life was still associated with depressive symptoms (β = −0.46, *p* < 0.001, 95%CI = −0.67, −0.25) in model b, while search for life was not associated with depressive symptoms (β = −0.04, *p* > 0.05, 95%CI = −0.23, −0.14) in model d. The interaction between WPV and presence of life had a significant effect on depressive symptoms (β = −0.23, *p* < 0.01, 95%CI = −0.40, −0.06) in model b. It proved that presence of life moderated the relationship between WPV and depressive symptoms. The interaction between WPV and search for life had a nonsignificant effect on depressive symptoms (β = −0.12, *p* > 0.05, 95%CI = −0.27, 0.04) in model d.

### 3.3. The Moderating Model between WPV, Meaning in Life, Presence of Life and Depressive Symptoms

**Figure 1:**
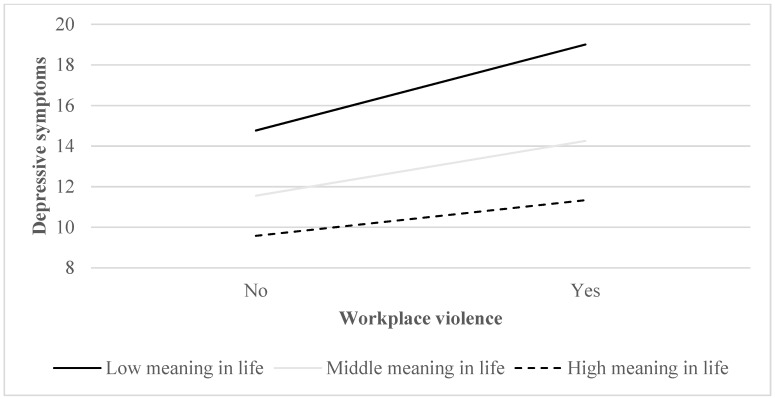
Meaning in life moderated the relationship between workplace violence and depressive symptoms among primary health care professionals.

The moderating analysis shown in Figure 1 was conducted utilizing PROCESS to identify the moderating role of meaning in life. When the moderating value was smaller than 41, as low meaning in life, the correlation between WPV and depressive symptoms grew more positive. When the moderating value was larger than 62, as high meaning in life, the correlation between WPV and depressive symptoms grew less positive.

**Figure 2:**
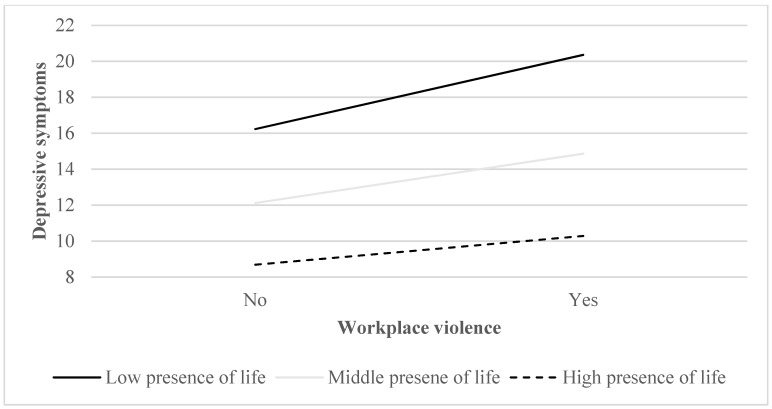
Presence of life moderated the relationship between workplace violence and depressive symptoms among primary health care professionals.

Figure 2 showed the moderating role of presence of life. When the moderating value was smaller than 20, as low presence of life, the correlation between WPV and depressive symptoms grew more positive. When the moderating value was larger than 31, high presence of life, the correlation between WPV and depressive symptoms grew less positive.

In summary, the results showed that there were stronger associations between WPV and depressive symptoms with low presence of life than that with high presence of life. It meant that primary healthcare professionals with higher presence of life would soften their levels of depressive symptoms than those with lower presence of life.

## 4. Discussion

The current study aimed to investigate the relationship between WPV and depressive symptoms and the moderating role of meaning in life on the relationship between WPV and depressive symptoms among primary healthcare professionals in China. Primary healthcare professionals who experienced WPV had more depressive symptoms in China. As expected, meaning in life, as a protective moderator, accounted for the as-mentioned relationship, and only presence of life moderated the relationship.

The results in the present study are as follows: 15.7% of primary healthcare professionals experienced WPV by patients or their relatives, 14.9% of primary healthcare professionals experienced verbal abuse, 3.1% of primary healthcare professionals experienced physical assault, and 2.3% of the primary healthcare professionals experienced both of the above types perpetrated by patients or their relatives. Compared with past research, we could assert that the prevalence of WPV was lower in this study than in other studies. One reason might be that different measurements were used. Another might be that we limited the perpetrators, only including patients and their relatives. However, other studies did not limit the identities and included patients, their relatives, co-workers, and supervisors.

In this study, we found that WPV was associated with age, working days, working days/week, education, major, budget post, nature of job, and income level. Primary healthcare professionals with lower age, fewer years of employment, and fewer working days every week increased the risk of WPV. The results were similar to previous studies, which illustrated that they were not familiar with the new jobs and tried to adapt their jobs, so they did not have enough time to explore the surrounding situations or address the problems [15]. Previous studies found that young workers had a high risk of confronting WPV, and workers of different ages had various views and responses to WPV [33]. Primary healthcare professionals with higher degrees had a higher level of WPV than those with lower degrees. Primary healthcare professionals graduating from non-clinical medicine could have worse professional knowledge, and it was difficult for them to correctly diagnose and treat the rural patients. Budget post was a guarantee for healthcare workers, primary healthcare professionals with it would have stable salaries and jobs, and they did not endeavor to work without active impetus as same as those with low-income levels. There was a lack of resources and advanced devices in village clinics, so primary healthcare professionals could not treat illness, leading to WPV. The finding that WPV was not correlated with gender was consistent with previous studies [34].

Similar to the previous studies, WPV was positively correlated with depressive symptoms among primary healthcare professionals in China. Previous studies indicated that township general practitioners and nurses who experienced WPV were prone to depression [6]. Among other healthcare workers, the effects of WPV on physicians and nurses were significantly negative [35,36,37]. Studies found that physicians and nurses exposed to WPV were likely to be depressed and anxious using a propensity score-matching measure [11]. Females were more likely to develop depressive symptoms than males, and the finding was in line with the previous studies [38,39]. A low-income level was also significantly associated with depressive symptoms comparing with the general level of income, primary healthcare professionals with lower level of income were likely to be depressive. A high level of income was not significantly correlated with depressive symptoms.

We found that meaning in life, including presence of life and search for life, were negatively associated with depressive symptoms; the result was consistent with the previous studies. The previous literature showed that meaning in life was correlated with depressive symptoms, anxiety, and stress [24]. A previous study revealed that lack of meaning in life was both an origin and impact of depression [40]. Primary healthcare professionals with higher meaning in life were less depressed than those with lower meaning in life. Existing studies showed that meaning in life could alleviate suicide ideation [27] and distress [41], as well as improving well-being [42] and cognitive functioning [25]. Meaning in life and presence of life were also negatively associated with WPV. Primary healthcare professionals exposed to WPV had lower meaning in life and presence of life than those who were not exposed to WPV. Primary healthcare professionals who confronted WPV by patients or their relatives perceived that they were not trusted, their jobs were meaningless, and did not help their patients, leading to a low level of meaning in life and mental problems such as depressive symptoms.

As expected, we found that meaning in life and presence of life moderated the relationship between WPV and depressive symptoms among primary healthcare professionals. Search for life did not moderate the aforementioned relationship. For primary healthcare professionals with high presence of life, presence of life lessened the relationship between WPV and depressive symptoms. For primary healthcare professionals with low presence of life, presence of life conversely aggravated the relationship. Presence of life might help primary healthcare professionals comprehend their jobs, ensure their purpose, control their emotions well, and pursue worthy ideals, leading to lower level of depression [43,44]. In other words, primary healthcare professionals who understood the unbearable suffering with clear and stable presence of life were better able to protect against adverse overcomes.

WPV is not only a common but also a profound social problem for healthcare workers. Improving workplace safety and health is also a challenging task for institutions [45]. Firstly, the administrators should adopt measures to prevent and monitor the incidents of WPV and provide intervention projects to decrease depressive symptoms, as well as training programs which increase their presence of life. Higher level of presence of life enable primary healthcare professionals to move easily after WPV rather than sinking into the violence [26]. On a second level, we suggest that the government launches policies to improve subsidies and pensions and raise the income and status among primary healthcare professionals, increasing job confidence, job satisfaction, and primary healthcare quality under the advocacy of the government. Promoting the physician–patient relationship is also equally crucial.

There are limitations in the present study. On the one hand, this is a cross-sectional study using self-reported questionnaires. The casual relationship is uncertain, and it is possible that WPV influences depressive symptoms, while depressive symptoms may lead to WPV. In the future, there is a need for a longitudinal study to clarify the findings in this study. On the other hand, the participants are all primary healthcare professionals in Shandong province, China. The findings may not be generalizable; wider ranges of participants may represent the generalizability of the study and results. At the same time, we have limited the identities of perpetrators and types of WPV, which may lead to lower incidences of WPV being reported. In addition, there are other factors that may influence meaning in life and depressive symptoms, such as life and job satisfaction. Future studies should consider these possible factors in the moderating models. However, the strengths exceed the limitations in the current study, providing insights for future research.

## 5. Conclusions

The current study has verified the association between WPV and meaning in life and the relationship between WPV and depressive symptoms among primary healthcare professionals in China. The findings also suggest that meaning in life and presence of life as moderators play crucial roles in the aforementioned relationship. Presence of life may decrease the effects of WPV on depressive symptoms; contrary, lack of presence of life may increase the influences of WPV on depressive symptoms among primary healthcare professionals.

## Figures and Tables

**Table 1:** Descriptive analysis and differences between primary healthcare professionals experienced workplace violence or not. [M ± SD/N (%)].

Variables	Total	WPV		*F*/*χ*^2^
		Yes	No	
N	2530	398 (15.7)	2132 (84.3)	
Age	41.55 ± 0.80	39.12 ± 9.00	42.01 ± 9.87	29.372 ***
Gender				0.393
Male	1089 (43.0)	177 (16.3)	912 (83.7)	
Female	1441 (57.0)	221 (15.3)	1220 (84.7)	
Religious belief				0.792
Yes	118 (4.7)	22 (18.6)	96 (81.4)	
No	2412 (95.3)	376 (15.6)	2036 (84.4)	
Working years	19.67 ± 12.02	16.11 ± 10.74	20.34 ± 12.13	42.252 ***
Working days/week	6.40 ± 0.87	6.06 ± 0.84	6.35 ± 0.86	38.037 ***
Education				63.424 ***
High school and above	842 (33.3)	194 (23.0)	648 (77.0)	
Technical school	915 (36.2)	137 (15.0)	778 (85.0)	
Technical secondary school and below	773 (30.6)	67 (8.7)	706 (91.3)	
Major				8.522 **
Clinical medicine	1276 (50.4)	174 (13.6)	1102 (86.4)	
Others	1254 (49.6)	224 (17.9)	1030 (82.1)	
Budgeted post				10.462 **
Yes	1005 (39.7)	179(17.8)	826(82.2)	
No	1439 (56.9)	214(14.9)	1225(85.1)	
Unknown	86 (3.4)	5(1.3)	81(94.2)	
Nature of job				9.273 **
Township hospitals	1454 (57.4)	201 (13.8)	1252 (86.2)	
Village clinics	1077 (42.6)	197 (18.3)	880 (81.7)	
Job types				3.543
Medicine	870 (34.4)	147 (16.9)	723 (83.1)	
Nursing	381 (15.1)	49 (12.9)	332 (87.1)	
Public health	911 (36.0)	147 (16.1)	764 (83.9)	
Others	368 (14.5)	55 (14.9)	313 (85.1)	
Income level (colleague)				7.513 *
Higher	320 (12.6)	45(14.1)	275(85.9)	
Average	1435 (56.7)	208(14.5)	1227(57.6)	
Lower	775 (30.6)	145(18.7)	630(81.3)	
Depressive symptoms	12.64 ± 9.71	15.94 ± 11.00	12.02 ± 9.32	55.765 ***
Meaning in life	51.61 ± 10.41	50.24 ± 10.39	51.87 ± 10.40	8.292 **
Presence of life	25.93 ± 5.40	26.09 ± 5.36	25.05 ± 5.52	12.533 ***
Search for life	25.69 ± 6.66	25.78 ± 6.69	25.19 ± 6.47	2.658

Note: *: *p* < 0.05; **: *p* < 0.01; ***: *p* < 0.001. WPV: workplace violence; M: Mean; SD: standard deviation.

**Table 2:** Linear regressions for the variables associated with depressive symptoms among primary healthcare professionals [β (95% CI)].

Variables	Model 1	Model 2
Age	−0.09 (−0.18, <0.001)	−0.08 (−0.17, 0.01)
Female	−2.00 (−3.12, −0.86) **	−1.95 (−3.08, −0.83) ***
Religious belief	1.40 (−0.27, 3.08)	1.35 (−0.33, 3.02)
Working years	0.03 (−0.05, 0.11)	0.03 (−0.06, 0.11)
Working days/week	−0.12 (−0.61, 0.37)	−0.12 (−0.60, 0.37)
Education (Ref. = Technical secondary school and below)		
High school and above	−0.02 (−1.11, 1.07)	−0.06 (−1.09, 1.08)
Technical school	−0.42 (−1.37, 0.53)	−0.40 (−1.34, 0.55)
Clinical medicine	0.55 (−0.29, 1.39)	0.55 (−0.30, 1.39)
Budgeted posts (Ref. = Unknown)		
Yes	1.39 (−0.68, 3.45)	1.37 (−0.70, 3.45)
No	1.15 (−0.85, 3.15)	1.15 (−0.85, 3.15)
Township hospitals	0.15 (−1.25, 1.56)	0.20 (−1.20, 1.60)
Job types (Ref. = Others)		
Medicine	0.42 (−0.80, 1.63)	0.46 (−0.76, 1.67)
Nursing	1.01 (−0.29, 2.31)	1.03 (−0.27, 2.32)
Public health	0.19 (−1.00, 1.37)	0.19 (−1.00, 1.37)
Income level (Ref. = Average)		
Higher	0.14 (−0.98, 1.26)	0.19 (−0.92, 1.31)
Lower	2.73 (1.91, 3.55) ***	2.71 (1.89, 3.53) ***
WPV	3.11 (2.12, 4.11) ***	9.09 (4.26, 13.91) ***
Meaning in life	−0.27 (−0.30, −0.23) ***	−0.13 (−0.24, −0.02) *
WPV × Meaning in life	––	−0.12 (−0.21, −0.03) *
Constant	25.04 (19.58, 30.49) ***	17.82 (9.93, 25.71) ***
R^2^	0.14	0.15

Note: β was the unstandardized regression coefficient; CI denotes to confidence interval; WPV denotes to workplace violence. –– denotes to the variable is not considered in Model. *: *p* < 0.05; **: *p* < 0.01; ***: *p* < 0.001.

**Table 3:** Linear regressions for presence of life and search for life associated with depressive symptoms among primary healthcare professionals [β (95% CI)].

Variables	Model a	Model b	Model c	Model d
WPV	2.926 (1.98, 3.87) ***	8.72 (4.37, 13.07) ***	3.42 (2.39, 4.45) ***	6.41 (2.36, 10.46) **
Presence of life	−0.72 (−0.79, −0.67) ***	−0.46 (−0.67, −0.25) ***	––	––
WPV × Presence of life		−0.23 (−0.40, −0.06) **	––	––
Search for life	––	––	−0.18 (−0.23, −0.12) ***	−0.04 (−0.23, 0.14)
WPV × Search for life	––	––	––	−0.12 (−0.27, 0.04)
Constant	28.50 (23.35, 33.65) ***	21.57 (14.34, 28.80) ***	16.31 (10.74, 21.88) ***	12.65 (5.31, 19.99) **
R^2^	0.47	0.47	0.28	0.28

Note: β was the unstandardized regression coefficient; CI denotes to confidence interval; WPV denotes to workplace violence. **: *p* < 0.01; ***: *p* < 0.001. Controlling for age, gender, religious belief, working years, working days/week, education, major, budget post, nature of job, job types, and income level. –– denotes to the variable is not considered in Model.

## Data Availability

Data are available from the corresponding author Long Sun upon reasonable request and with permission of IRB of Shandong University School of public health, which were used under license for the current study, and so are not publicly available.
